# Derived neutrophil-to-lymphocyte ratio has the potential to predict safety and outcomes of durvalumab after chemoradiation in non-small cell lung cancer

**DOI:** 10.1038/s41598-024-70214-y

**Published:** 2024-08-23

**Authors:** Akira Sugimoto, Hiroyasu Kaneda, Naoki Yoshimoto, Kenji Nagata, Tatsuo Fujii, Koichi Michimoto, Shunsuke Ueno, Takao Kamimori, Yoshie Ishii, Mai Sakagami, Haruo Inokuchi, Keiko Shibuya, Megumi Mizutani, Hiroaki Nagamine, Kenji Nakahama, Yoshiya Matsumoto, Yoko Tani, Kenji Sawa, Tomoya Kawaguchi

**Affiliations:** 1 Department of Respiratory Medicine, Graduate School of Medicine, Osaka City University, 1-4-3 Asahi-machi, Abeno-ku, Osaka, 545-8585 Japan; 2 Department of Respiratory Medicine, Graduate School of Medicine, Osaka Metropolitan University, 1-4-3 Asahi-machi, Abeno-ku, Osaka, 545-8585 Japan; 3 Department of Clinical Oncology, Graduate School of Medicine, Osaka Metropolitan University, 1-4-3 Asahi-machi, Abeno-ku, Osaka, 545-8585 Japan; 4 Department of Respiratory Medicine, Ishikiriseiki Hospital, 18-28 Yayoi-cho, Higashiosaka, Osaka 579-8026 Japan; 5 Department of Radiation Oncology, Ishikiriseiki Hospital, 18-28 Yayoi-cho, Higashiosaka, Osaka 579-8026 Japan; 6 Department of Respiratory Medicine, Osaka General Hospital of West Japan Railway Company, 1-2-22 Matsuzaki-cho, Abeno-ku, Osaka, 545-0053 Japan; 7 Department of Radiation Therapy, Osaka General Hospital of West Japan Railway Company, 1-2-22 Matsuzaki-cho, Abeno-ku, Osaka, 545-0053 Japan; 8 Department of Respiratory Medicine, Yodogawa Christian Hospital, 1-7-50 Kunijima, Higashiyodogawa-ku, Osaka, 533-0024 Japan; 9 Department of Radiation Therapy, Yodogawa Christian Hospital, 1-7-50 Kunijima, Higashiyodogawa-ku, Osaka, 533-0024 Japan; 10 Department of Radiation Oncology, Graduate School of Medicine, Osaka Metropolitan University, 1-4-3 Asahi-machi, Abeno-ku, Osaka, 545-8585 Japan

**Keywords:** Durvalumab, Non small cell lung cancer, Derived neutrophil to lymphocyte ratio, Pneumonitis, Immune related adverse event, Biomarkers, Oncology, Lung cancer

## Abstract

The usefulness of the derived neutrophil-to-lymphocyte ratio (dNLR) and its dynamics before/after durvalumab consolidation therapy to predict safety or efficacy remains unclear. We retrospectively reviewed patients with locally advanced non-small cell lung cancer treated with durvalumab consolidation therapy after chemoradiotherapy (D group) or chemoradiotherapy alone (non-D group) at multiple institutions. We investigated the association between dNLR, or its dynamics, and pneumonitis, checkpoint inhibitor-related pneumonitis (CIP), irAEs, and efficacy. Ninety-eight and fifty-six patients were enrolled in the D and non-D groups, respectively. The dNLR at baseline was significantly lower in patients who experienced irAEs or CIP than in those who did not. The low dNLR group, 28 days following durvalumab consolidation therapy (dNLR28 ≤ 3), demonstrated longer progression-free survival (PFS) and overall survival (OS) than the high dNLR group (dNLR28 > 3) (PFS, hazard ratio [HR] 0.44, 95% confidence interval [CI] 0.22–0.88, *p* = 0.020; OS, HR 0.39, 95% CI 0.16–0.94, *p* = 0.037). Among patients with high dNLR at baseline (dNLR > 3), the dNLR28 ≤ 3 group showed longer PFS than the dNLR28 > 3 group (*p* = 0.010). The dNLR is a predictive factor for irAEs and CIP in patients receiving durvalumab consolidation therapy. The dNLR at 28 days after durvalumab consolidation therapy and its dynamics predict favorable outcomes.

## Introduction

Durvalumab consolidation therapy after chemoradiotherapy (CRT) has improved the survival of patients with unresectable locally advanced non-small cell lung cancer (NSCLC)^1,2^. However, immune checkpoint inhibitors (ICIs) can cause severe immune-related adverse events (irAEs)^3^. Pneumonitis is a common adverse event that develops during durvalumab consolidation therapy after CRT in patients with unresectable locally advanced NSCLC that sometimes requires systemic corticosteroid therapy and leads to treatment discontinuation or death^1^. Previous research suggests that dosimetric parameters of radiation therapy, such as mean lung dose (MLD) and lung volume receiving a dose of ≥ 20 Gy (V20), are risk factors of pneumonitis in patients treated with durvalumab consolidation therapy after CRT^4–6^. However, predictive markers for risk factors of pneumonitis in durvalumab after CRT have not been established except for radiation-related parameters. Thus, exploring the predictive factors for irAEs and systemic corticosteroid-required or severe pneumonitis is significant.

The neutrophil-to-lymphocyte ratio (NLR) predicts the efficacy and safety of ICIs in various cancers^7–12^. Traditional NLR is the marker evaluated using neutrophils and lymphocytes. However, irAEs and efficacy of ICIs were affected by neutrophils and lymphocytes as well as monocytes and other granulocytes^13,14^. In contrast, the derived NLR (dNLR) is calculated by dividing neutrophil counts by total white blood cell count—neutrophil count^15^. Therefore, dNLR may reflect a more comprehensive immune status because dNLR can be evaluated, including neutrophils and lymphocytes as well as monocytes and other granulocytes^14^. We chose to use dNLR in our study because dNLR is a more robust marker in settings where lymphocyte levels might be affected by various factors to more accurately assess the inflammatory state. dNLR is also a predictive marker for the efficacy of ICIs in patients with advanced NSCLC^16^. In durvalumab consolidation therapy following CRT for unresectable locally advanced NSCLC, previous studies have reported the usefulness of the NLR for predicting efficacy^17,18^. Nevertheless, the significance of the dNLR as a predictive marker of efficacy and safety especially regarding pneumonitis and irAEs in durvalumab consolidation therapy after CRT remains unclear. Moreover, it is important to evaluate the change in NLR from baseline since the NLR changes during the treatment^17,19^. However, the utility of dNLR dynamics after the initiation of durvalumab consolidation therapy has rarely been reported. The predictive value of dNLR dynamics for the efficacy and safety of durvalumab consolidation therapy is also unclear.

Herein, we evaluated the significance of dNLR before or after durvalumab consolidation therapy as predictive factors for efficacy and safety, especially pneumonitis or irAEs, in patients who received durvalumab consolidation therapy after CRT compared with those who received CRT only as a historical control.

## Materials and methods

### Patients

Patients were recruited from four hospitals (Osaka Metropolitan University Hospital, Ishikiriseiki Hospital, Osaka General Hospital of West Japan Railway Company, and Yodogawa Christian Hospital). Those who met the following inclusion criteria were enrolled in the durvalumab group (D group): (i) patients who received CRT for unresectable locally advanced NSCLC from August 2018 to March 2022, (ii) experienced no disease progression in the evaluation immediately after CRT, and (iii) received durvalumab consolidation therapy. Inclusion criteria for the non-durvalumab group (non-D group) as a historical control were as follows: (i) patients who received CRT alone at Osaka Metropolitan University Hospital from August 2014 to July 2018 and (ii) experienced no disease progression in the evaluation immediately after CRT. Similar to the PACIFIC trial, patients with grade ≥ 2 pneumonitis before the initiation of durvalumab consolidation therapy in the D group and within 42 days after the completion of radiation therapy in the non-D group were excluded^1^. All patients, except those who were not followed up after CRT, were enrolled in the efficacy analysis dataset. Of these, patients without dosimetric data of radiation therapy were excluded from the safety analysis dataset.

This study was approved by the institutional review boards (numbers: 2022–145 for Osaka Metropolitan University Hospital, Ishikiriseiki Hospital, and Yodogawa Christian Hospital and 2022–17 for Osaka General Hospital of West Japan Railway Company). Informed consent was obtained from patients using an opt-out form based on the Ethical Guidelines for Medical and Biological Research Involving Human Subjects by the Japanese Ministry of Health, Labour and Welfare to provide a chance to decline participation. All methods were performed in accordance with the relevant guidelines and regulations.

### Data collection

Clinical data were retrospectively collected from medical records. Clinical data included age, sex, smoking history, Eastern Cooperative Oncology Group performance status (ECOG PS), emphysema, stage (according to the 8th edition of the tumor-node-metastasis classification of the Union for International Cancer Control/the American Joint Committee on Cancer), histology, epidermal growth factor receptor (*EGFR*) mutation status, anaplastic lymphoma kinase (*ALK*) fusion status, programmed death-ligand 1 (PD-L1) expression (22C3), chemotherapy regimen, dosimetric data of radiation therapy, survival data, adverse events, and blood test data. Adverse events after CRT considered treatment-related were collected and evaluated based on the Common Terminology Criteria for Adverse Events version 5.0.

### Evaluation of pneumonitis

Radiation pneumonitis or checkpoint inhibitor-related pneumonitis (CIP) was assessed by physicians based on radiographic findings such as X-ray or computed tomography (CT) images, radiologists’ reports, and other clinical information including clinically recommended serologic, immunologic, and histologic testing.

### Definition of dNLR

The dNLR was calculated as follows:

Absolute neutrophil count/(white blood cell count—absolute neutrophil count)^15^.

In the D group, dNLR was calculated at the initiation of durvalumab consolidation therapy at baseline and 28 days (defined as dNLR28) after the initiation of durvalumab consolidation therapy. In the non-D group, dNLR was calculated at the first outpatient visit after radiation therapy completion at baseline and then at 28 days (dNLR28) after the first outpatient visit. Blood data for calculating dNLR28 were from 1 week before and after 28 days.

For survival analysis, dNLR and dNLR28 were divided into two groups (≤ 3, > 3) based on previous studies^16,20^.

### Statistical analyses

Patient characteristics were analyzed using Fisher’s exact test for categorical variables and the Wilcoxon rank-sum test for continuous variables. The relationship between dNLR and pneumonitis, CIP, or irAE was analyzed using the Wilcoxson sum rank test. The cumulative incidence of pneumonitis was evaluated using Gray’s test. Univariable and multivariable analyses of the survival data, including the association of dNLR and efficacy of durvalumab consolidation therapy, were performed using the Cox proportional hazards model. Survival curves were documented using the Kaplan–Meier method and analyzed using the log-rank test. For survival analysis using dNLR on days 28 after the initiation of durvalumab consolidation therapy, landmark analyses were performed for patients who experienced no disease progression and continued the therapy on day 28 from the initiation of the therapy in the D group or patients without disease progression on day 28 from the first outpatient visit after the completion of radiation therapy in the non-D group. A *p*-value < 0.05 was considered statistically significant. Statistical analyses were performed using the EZR software (Saitama Medical Center, Jichi Medical University, Japan)^21^.

## Results

### Patient characteristics

Ninety-eight patients were enrolled in the D group (Supplementary Fig. 1). One patient who was not followed up after the initiation of durvalumab consolidation therapy and one who had grade 2 pneumonitis before the initiation of durvalumab consolidation therapy were excluded from the efficacy and safety analysis datasets. Fifty-six patients were enrolled in the non-D group. Four patients who were not followed up after CRT and four patients who developed grade 2 pneumonitis within 42 days of radiation therapy completion were excluded from the efficacy analysis dataset. Of these, 16 patients with no dosimetric data for radiation therapy were excluded from the safety analysis dataset. The efficacy analysis dataset included 96 and 48 patients in the D and non-D groups, respectively. The safety analysis dataset comprised 96 and 32 patients in the D and non-D groups, respectively.

Patient characteristics are shown in Table 1. The median ages were 69 and 67 years in the D and non-D groups, respectively. Characteristics, including sex, smoking history, ECOG PS, emphysema, stage, and histology, were not noticeably different between the D and non-D groups. Although *EGFR* mutation and *ALK* fusion status were unknown in most patients, six and four patients in the D and non-D groups, respectively, had *EGFR* mutations, and two in the D group had *ALK* fusions. PD-L1 expression was evaluated in only 9 (18.8%) patients in the non-D group. In contrast, in the D group, PD-L1 expression was evaluated in 77 patients. Of these, 28 (29.2%) had PD-L1 < 1%, and 16 (16.7%) had PD-L1 ≥ 50%. In both groups, the most common chemotherapy regimen for CRT was carboplatin plus paclitaxel. Almost all patients in both groups received concurrent radiotherapy. The median radiation dose was 60 Gy in both groups. The dosimetric data for radiotherapy are presented in Supplementary Table 1. In the D group, 21 (21.9%) patients were treated using intensity-modulated radiotherapy. More patients in the D group underwent involved-field radiation therapy (IFRT) and not elective nodal irradiation (ENI), whereas more patients in the non-D group underwent ENI and not IFRT. However, there were no significant differences in the mean lung dose (MLD), V5, V20, and VS5.

**Table 1:** Patient characteristics.

Factor		non-D group (n = 48)	D group (n = 96)	*p*-value
Age, median (range), year		67 (42–83)	69 (42–82)	0.305
Sex, n (%)	Male	34 (70.8)	65 (67.7)	0.849
	Female	14 (29.2)	31 (32.3)	
Smoking history, n (%)	Never	4 (8.3)	18 (18.8)	0.211
	Former	21 (43.8)	32 (33.3)	
	Current	23 (47.9)	46 (47.9)	
ECOG PS, n (%)	0	21 (43.8)	37 (38.5)	0.591
	1	27 (56.2)	59 (61.5)	
Emphysema, n (%)	No	15 (31.2)	39 (40.6)	0.361
	Yes	33 (68.8)	57 (59.4)	
Stage, n (%)	IIB	1 (2.1)	2 (2.1)	0.826
	IIIA	23 (47.9)	40 (41.7)	
	IIIB	20 (41.7)	41 (42.7)	
	IIIC	4 (8.3)	13 (13.5)	
Histology, n (%)	Squamous	21 (43.8)	44 (45.8)	0.968
	Adeno	22 (45.8)	41 (42.7)	
	Other	5 (10.4)	11 (11.5)	
EGFR mutations, n (%)	None	16 (33.3)	51 (53.1)	0.082
	Yes	4 (8.3)	6 (6.2)	
	Unknown	28 (58.3)	39 (40.6)	
ALK fusions, n (%)	None	15 (31.2)	50 (52.1)	0.019
	Yes	0 (0.0)	2 (2.1)	
	Unknown	33 (68.8)	44 (45.8)	
PD-L1 (22C3), n (%)	< 1%	1 (2.1)	28 (29.2)	< 0.001
	1–49%	4 (8.3)	33 (34.4)	
	≧ 50%	4 (8.3)	16 (16.7)	
	Unknown	39 (81.2)	19 (19.8)	
Chemotherapy in CRT, n (%)	CDDP + VNR	17 (35.4)	17 (17.7)	0.009
	CBDCA + PTX	25 (52.1)	74 (77.1)	
	Daily CBDCA	3 (6.2)	1 (1.0)	
	CDDP + S − 1	0 (0.0)	1 (1.0)	
	Other	3 (4.2)	3 (3.1)	
Radiation therapy, n (%)	Concurrent	48 (100.0)	94 (97.9)	0.552
	Sequential	0 (0.0)	2 (2.1)	
Radiation dose, median (range), Gy		60 (56–66)	60 (56–70)	0.003

non-D group, non-durvalumab group; D group, durvalumab group; ECOG PS, Eastern Cooperative Oncology Group performance status; EGFR, epidermal growth factor receptor; ALK, anaplastic lymphoma kinase; PD-L1, programmed death-ligand 1; CRT, chemoradiotherapy; CDDP, cisplatin; VNR, vinorelbine; CBDCA, carboplatin; PTX, paclitaxel, S-1, tegafur/gimeracil/oteracil.

The median duration of durvalumab consolidation therapy was 7.0 (range, 0.03–13.1) months. The median number of cycles of durvalumab consolidation therapy was 13.5 (range, 1–27) cycles. Among the 96 patients, 36 completed durvalumab consolidation therapy for 1 year, 7 were on treatment, 23 experienced disease progression, 25 discontinued because of adverse events, and 5 discontinued owing to other reasons.

### Adverse events

Adverse events of any grade were observed in 89 (92.7%) and 30 (93.7%) patients in the D and non-D groups, respectively. The most common adverse events were pneumonitis (92.7%), hypothyroidism (10.4%), rash (8.3%), and infusion reaction (6.2%) in the D group and pneumonitis (93.7%) and malaise (9.3%) in the non-D group. irAEs occurred in 38 (39.5%) patients in the D group. The most frequent irAEs were pneumonitis (11.4%) and hypothyroidism (10.4%), rash (8.3%), and infusion reaction (6.2%) (Supplementary Table 2). Among these, 14 required systemic steroid therapy due to irAEs.

Regarding pneumonitis, in the D group, grade ≥ 2 pneumonitis was observed in 34 (35.4%) patients, including 29 (30.2%) and 5 (5.2%) grades 2 and 3 pneumonitis, respectively. In the non-D group, 3 (grade 2, 2 patients; grade 5, 1 patient) patients experienced grade ≥ 2 pneumonitis (Supplementary Table 3). Among patients who experienced pneumonitis in each group, the number of those with pneumonitis within and beyond the irradiation field, respectively, was as follows: D group, 82 (92.1%), 7 (7.9%); non-D group, 29 (96.7%), 1 (3.3%). The cumulative incidence rate of grade ≥ 2 pneumonitis was significantly higher in the D group than in the non-D group (p < 0.01) (Supplementary Fig. 2). Half of the patients in the D group had a pneumonitis event within 2 months of starting durvalumab treatment. Among the 89 patients who had pneumonitis in the D group, 11 patients had CIP, and their severity was as follows: grade 1, 1; grade 2, 7; and grade 3, 3 (Supplementary Table 3). Pneumonitis requiring systemic corticosteroid therapy was observed in 34 and 3 patients in the D and non-D groups, respectively.

### Association between dNLR and pneumonitis

**Figure 1:**
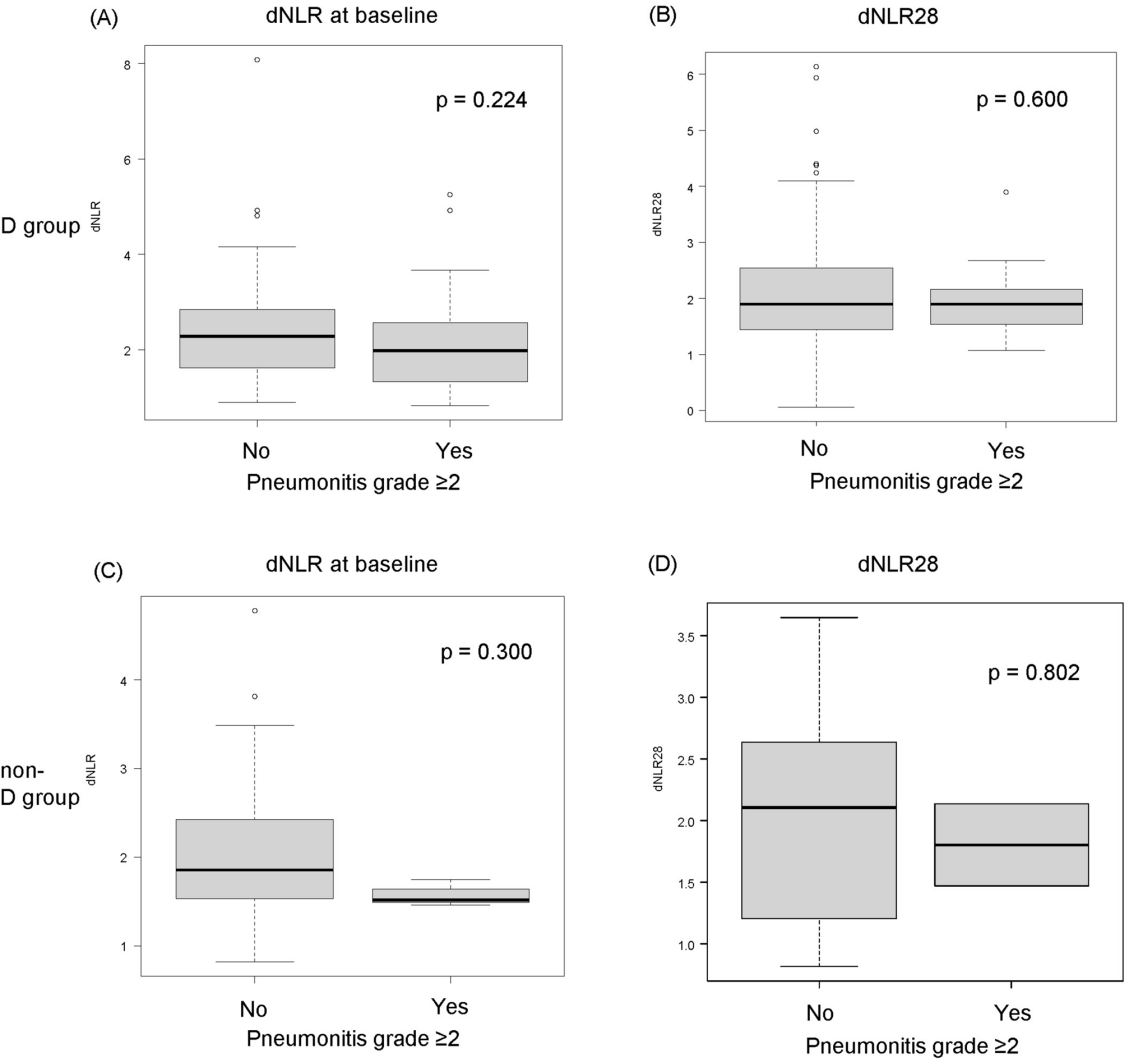
Association between dNLR and grade ≥ 2 pneumonitis. (**A**) dNLR, and (**B**) dNLR28 in patients who did and did not experience grade ≥ 2 pneumonitis in the D group. (**C**) dNLR, and (**D**) dNLR28 in patients who experienced grade ≥ 2 pneumonitis in the non-D group. dNLR, derived neutrophil-to-lymphocyte ratio; D group, durvalumab group; non-D group, non-durvalumab group.

In the D group, dNLR was not significantly different between patients with grade ≥ 2 pneumonitis and those without pneumonitis at baseline (Fig. 1A). On days 28 after the initiation of durvalumab consolidation therapy, dNLR was also not significantly different between patients with grade ≥ 2 pneumonitis and those without pneumonitis (Fig. 1B). In the univariable analysis, grade ≥ 2 pneumonitis was significantly associated with sex, V5, V20, and MLD but not with dNLR (Supplementary Table 4). The non-D group also did not differ significantly in dNLR both at baseline and day 28 between patients with grade ≥ 2 pneumonitis and those without pneumonitis (Fig. 1C and D). In the univariable analysis, grade ≥ 2 pneumonitis was significantly associated with the radiation method, but not with the dNLR (Supplementary Table 4).

Among patients who experienced pneumonitis in the D group, baseline dNLR was lower than in pneumonitis within the irradiation field than in that beyond the irradiation field (*p* = 0.020) (Supplementary Fig. 3). In the non-D group, the relationship between the area of pneumonitis could not be analyzed because there was only one patient with pneumonitis beyond the irradiation field.

**Figure 2:**
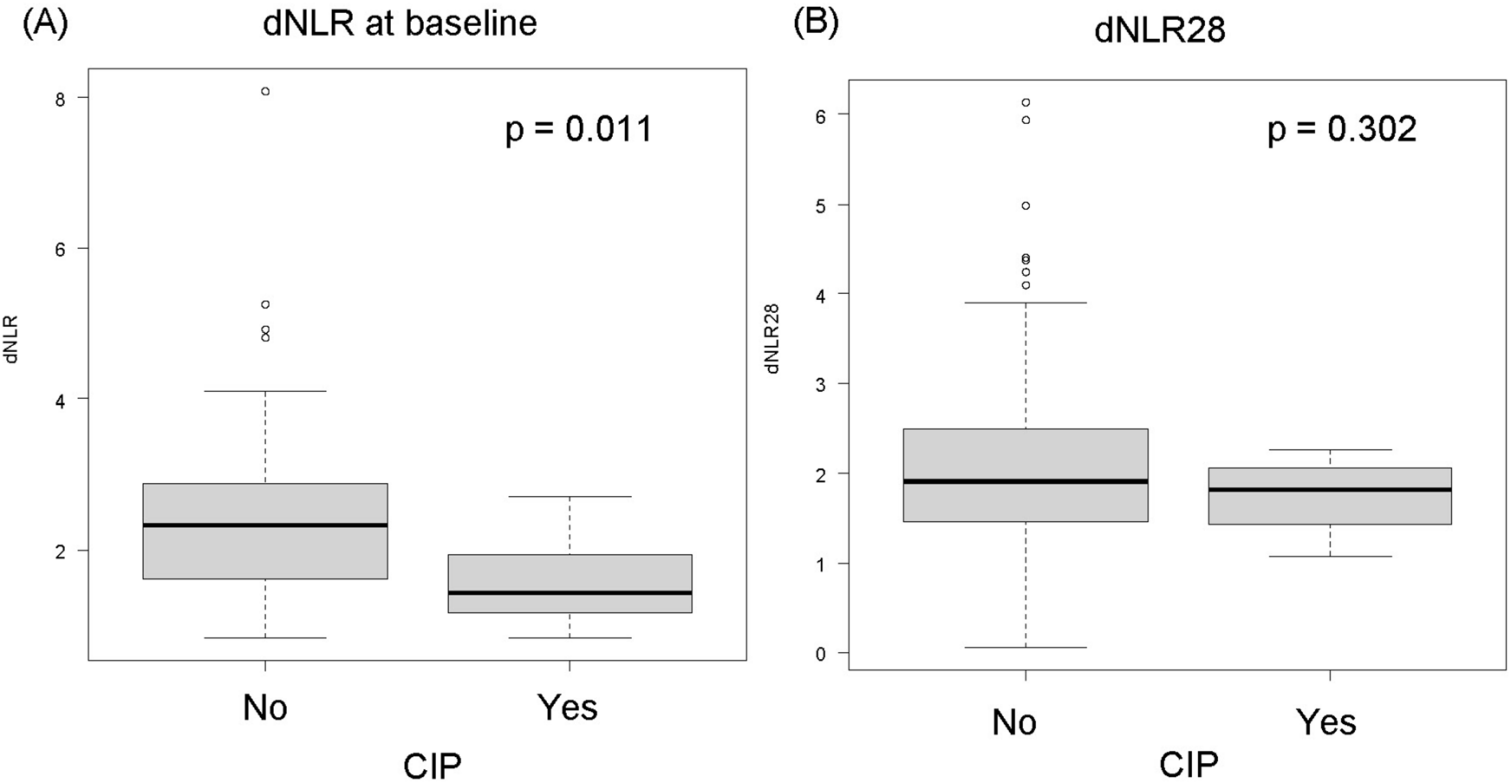
Association between dNLR and CIP. (**A**) dNLR, and (**B**) dNLR28 in patients who did and did not experience CIP derived neutrophil-to-lymphocyte ratio; CIP, checkpoint inhibitor-related pneumonitis.

At baseline, dNLR was significantly lower in patients who developed CIP than in those who did not (Fig. 2A). On days 28 after the initiation of durvalumab consolidation therapy, dNLR was not significantly different between patients who developed CIP and those who did not (Fig. 2B). In the univariable analysis, the incidence of CIP due to durvalumab was associated with low dNLR. (Supplementary Table 4).

### Association between dNLR and irAEs

dNLR at baseline was significantly lower in patients who developed irAEs than in those who did not at baseline in the D group (Supplementary Fig. 4A). On day 28 from the initiation of durvalumab consolidation therapy, dNLR was not significantly different between patients who developed irAEs and those who did not (Supplementary Fig. 4B). In the univariable and multivariable analyses, low dNLR was associated with irAEs (Supplementary Table 5).

### Treatment outcomes in patients with high or low dNLR among D group

**Figure 3:**
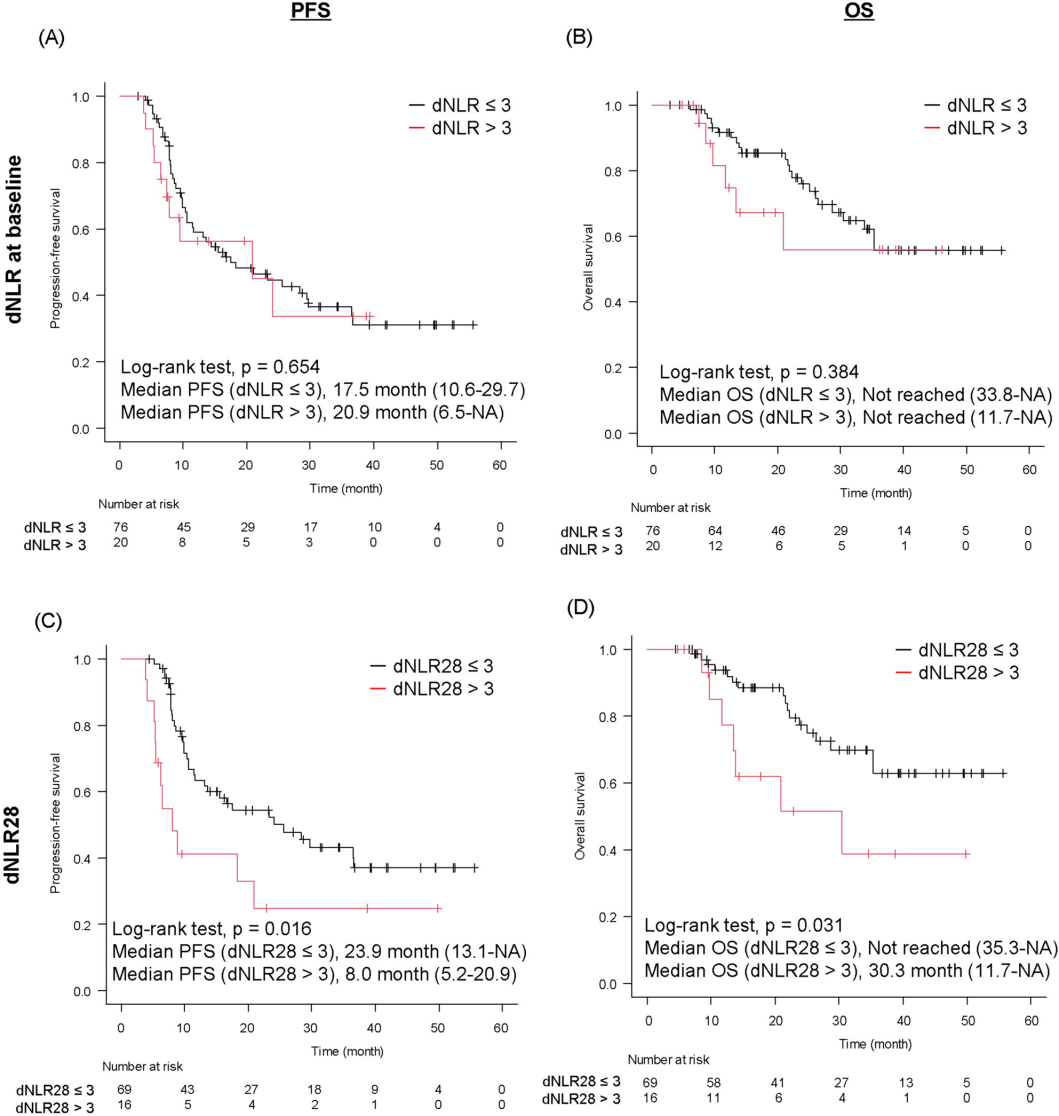
Treatment outcome according to dNLR value in the D group. (**A**) PFS and (**B**) OS in patients with dNLR ≤ 3 or > 3. (**C**) PFS and (**D**) OS in patients with dNLR28 ≤ 3 or > 3. dNLR, derived neutrophil-to-lymphocyte ratio; D group, durvalumab group; PFS, progression-free survival; OS, overall survival; NA, not applicable.

Among patients treated with durvalumab consolidation therapy, at baseline, the median progression-free survival (PFS) was similar (17.5 months [10.6 months to 29.7] vs. 20.9 months [6.5 months to not reached]) between the low dNLR and high dNLR groups (*p* = 0.654) (Fig. 3A). The median OS (not reached [33.8 months to not reached] vs. not reached [11.7 months to not reached]) was also similar between the two groups (*p* = 0.384) (Fig. 3B).

Four-week landmark analysis showed that dNLR28 was significantly associated with increased PFS and OS (Fig. 3C and D). The median PFS in the low dNLR group was significantly longer than that in the high dNLR group (23.9 months [13.1 months to not reached] vs. 8.0 [5.2 to 20.9] months; *p* = 0.016), and so was the median OS (not reached [35.3 months to not reached] vs. 30.3 [11.7 to not reached] months; *p* = 0.031).

We next analyzed the association of PFS and OS with patient characteristics (Supplementary Tables 6 and 7). The dNLR28 was significantly associated with better PFS in univariate and multivariate analysis (univariate, HR 0.44, 95%CI 0.22–0.88, *p* = 0.020; multivariate, HR 0.47, 95%CI 0.22–0.98, *p* = 0.044). Histology (non-squamous) and PD-L1 expression (≥ 50%) were also associated with better PFS in univariate analysis, and PD-L1 expression (≥ 50%) was associated with better PFS in multivariate analysis. The low dNLR28 was significantly associated with better OS in univariate analysis (HR 0.39, 95%CI 0.16–0.94, *p* = 0.037). In multivariate analysis, the low dNLR tended to be associated with better OS (HR 0.50, 95%CI 0.20–1.22, *p* = 0.131). Histology (non-squamous) was associated with better OS in univariate and multivariate analysis.

In contrast to the D-group, there was no association of dNLR at any point with PFS and OS in the non-D group. (median PFS: dNLR ≤ 3, 11.0 months, dNLR > 3, 4.2 months, *p* = 0.078; dNLR28 ≤ 3, 9.5 months, dNLR28 > 3, 9.2 months, *p* = 0.354; median OS: dNLR ≤ 3, not reached, dNLR > 3, not reached, *p* = 0.464; dNLR28 ≤ 3, 69.6 months, dNLR28 > 3, not reached, *p* = 0.614) (Supplementary Fig. 5A–D).

### Treatment outcomes according to changes in dNLR value in D-group

Among patients with baseline dNLR ≤ 3, at 28 days after starting durvalumab consolidation therapy, nine (13.4%) patients changed to dNLR > 3 (dNLR28 > 3) (defined as BL-low/28-high group) and 58 (86.6%) patients remained at dNLR ≤ 3 (dNLR28 ≤ 3) (defined as BL-low/28-low group) (Supplementary Fig. 6). Regarding patients with baseline dNLR > 3, 11 (61.1%) patients changed to dNLR ≤ 3 (dNLR28 ≤ 3) (defined as BL-high/28-low group) and seven (38.9%) patients remained at dNLR > 3 (dNLR28 > 3) (defined as BL-high/28-high group).

**Figure 4:**
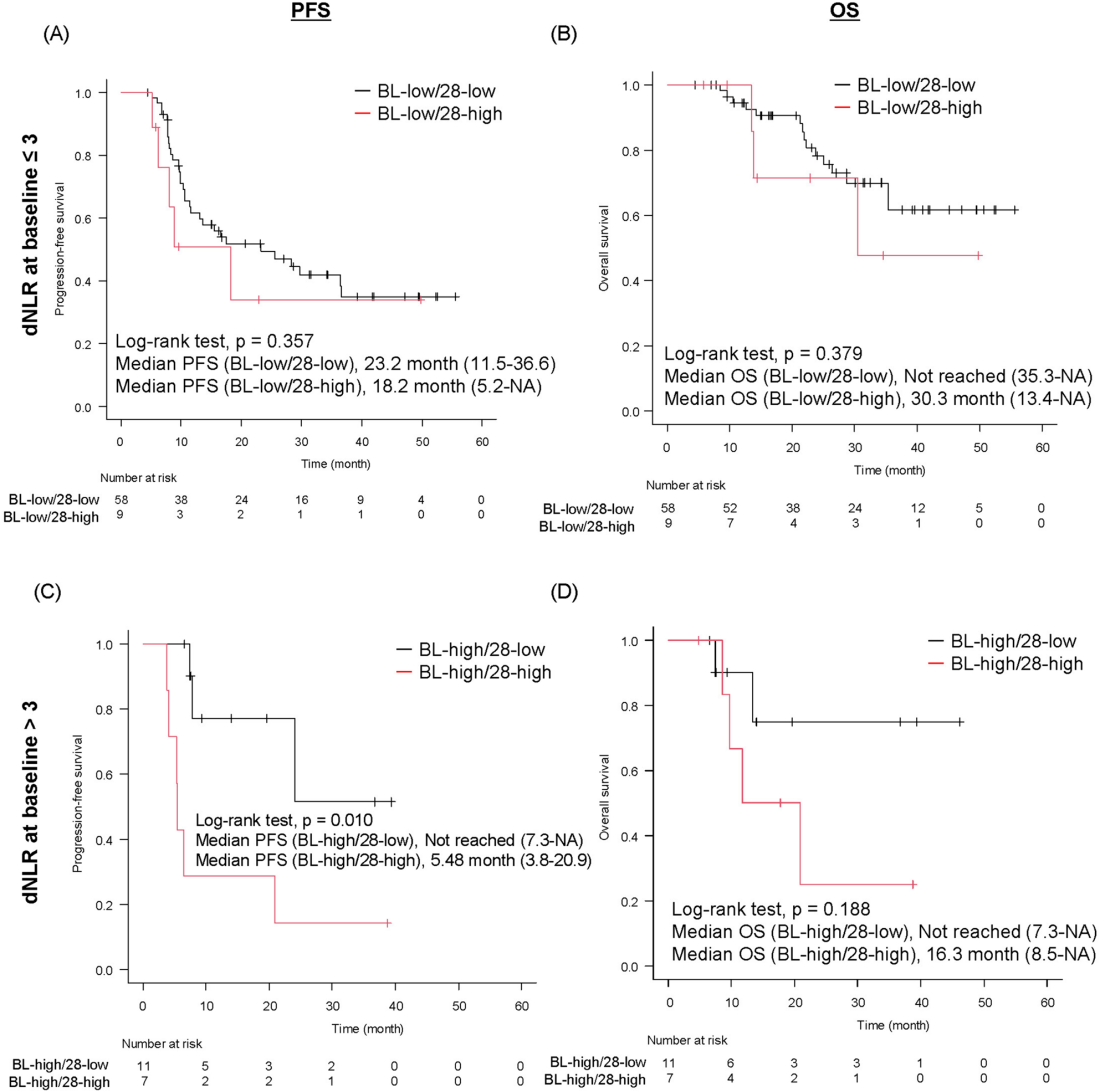
Treatment outcome according to changes in dNLR from baseline (BL) in D-group. (**A**) PFS (**B**) OS in patients of BL-low/28-low and BL-low/28-high groups. (**C**) PFS and (**D**) OS in patients with BL-high/28-low and BL-high/28-high groups. dNLR, derived neutrophil-to-lymphocyte ratio; D group, durvalumab group; PFS, progression-free survival; OS, overall survival; NA, not applicable; BL-low/28-low, patients with baseline dNLR ≤ 3 and dNLR28 ≤ 3; BL-low/28-high, patients with baseline dNLR ≤ 3 and dNLR28 > 3; BL-high/28-low, patients with baseline dNLR > 3 and dNLR28 ≤ 3; BL-high/28-low, patients with baseline dNLR > 3 and dNLR28 ≤ 3.

Among patients with baseline dNLR ≤ 3, both the median PFS (23.2 months [11.5 months to 36.6] vs. 18.2 months [5.2 months to not reached]; *p* = 0.357) (Fig. 4A) and the median OS (not reached [35.3 months to not reached] vs. 30.3 [13.4 to not reached] months; *p* = 0.379) (Fig. 4B) were similar between BL-low/28-low and BL-low/28-high groups. In contrast, in patients with baseline dNLR > 3, the BL-high/28-low group showed a significantly longer PFS and a tendency toward a longer OS than the BL-high/28-high group (median PFS: BL-high/28-low, not reached, BL-high/28-high, 5.48 months, *p* = 0.010; median OS: BL-high/28-low, not reached, BL-high/28-low, 16.3 months, *p* = 0.188) (Fig. 4C and D).

## Discussion

In our study, the dNLR at baseline and after the initiation of durvalumab consolidation therapy was not a predictor of grade ≥ 2 pneumonitis in patients with unresectable locally advanced NSCLC treated with durvalumab consolidation therapy after CRT. However, the baseline dNLR was a predictor of CIP and irAEs. Furthermore, a low dNLR 28 was associated with the favorable efficacy of durvalumab consolidation therapy but not associated with the survival in CRT alone group. Therefore, a low dNLR28 was not a prognostic factor but a predictive factor of durvalumab consolidation therapy. Interestingly, patients with a high dNLR at baseline (dNLR > 3), whose dNLR changed to ≤ 3 at 28 days after treatment initiation, had a favorable benefit of durvalumab consolidation therapy.

The cumulative incidence of grade ≥ 2 pneumonitis was higher in patients who received durvalumab consolidation therapy than those receiving CRT only. However, the incidence of all-grade and grade ≥ 3 pneumonitis was similar between groups. In the Japanese cohort of the PACIFIC trial, pneumonitis occurred in 73.6% of participants who received durvalumab for all grades, 7% for grade ≥ 3, 60% for those who received placebo for all grades, and 5% for grade ≥ 3. Two Japanese real-world studies reported that the incidence of all-grade pneumonitis in patients who received durvalumab was 84.9% and 85%^5,22^. These data are similar to our results. In the systematic review and meta-analysis, the incidence of all grades and grade ≥ 3 did not differ between CRT alone and CRT followed by durvalumab but that of grade ≥ 2 was higher for CRT followed by durvalumab^6^. Therefore, durvalumab consolidation therapy could increase the incidence of grade 2 pneumonitis. The PACIFIC trial reports a higher incidence of grade 2 pneumonitis in the durvalumab group than the placebo group (durvalumab, 15.2% [72/475]; placebo, 9.4% [22/234])^23^.

For the evaluation of pneumonitis, the predictive values of dNLR have not been established. Symptomatic pneumonitis occurs more frequently with a higher NLR in radiation pneumonitis (RP)^24^. According to Korean real-world data on durvalumab consolidation therapy, a high dNLR has a predictive value for RP requiring systemic corticosteroid therapy^25^. Pneumonitis during durvalumab consolidation therapy includes RP and CIP; CIP is an irAE. Lower NLR is associated with more frequent irAEs during ICI treatment for advanced NSCLC and other carcinomas^12,26^. There could be different NLR and dNLR between CIP and RP, resulting in differences in the predictive utility of dNLR for pneumonitis and CIP in our study. However, previous studies reported that NLR is not associated with CIP^27,28^. Although direct comparisons cannot be made since previous reports and our results were in different clinical scenarios, further validation of the usefulness of the dNLR as a predictor of CIP is required.

A high NLR is associated with poor efficacy of durvalumab consolidation therapy^29,30^. To the best of our knowledge, the utility of dNLR for predicting efficacy has not been reported in durvalumab consolidation therapy after CRT. However, in advanced NSCLC, a higher dNLR before ICI initiation is associated with poor efficacy of ICI treatment^16,31^. In this study, the dNLR before the initiation of durvalumab consolidation therapy was not a predictive factor of efficacy, but the dNLR 28 days after the initiation of durvalumab was. The dNLR before the initiation of durvalumab could be affected by myelosuppression caused by CRT, whereas at day 28, there was no effect of CRT-induced myelosuppression. This might have influenced the difference in the predictability of the dNLR before the initiation of durvalumab consolidation therapy and at 28 days post-treatment. Moreover, NLR and dNLR dynamics after treatment initiation are predictive of ICI treatment efficacy in advanced NSCLC^31–37^. It has been suggested that tumor microenvironments change in cases where the NLR or dNLR decreases after treatment, indicating that ICIs are effective. A previous study demonstrated that decreased NLR reflected the upregulation of interferon-γ response and antigen presentation and change of innate immune response gene signature^34^. NLR dynamics also reflect changes in the intratumoral T-cell repertoire. Another study suggested that decreasing dNLR in pembrolizumab treatment was associated with an increase in intratumoral CD8 + and PD-1 + immune cells resulting in a favorable microenvironment for ICI treatment^31^. These findings suggest that the dNLR at 28 days after the initiation of treatment is predictive of the efficacy of durvalumab consolidation therapy and patients whose dNLR changed from high to low have a favorable prognosis.

This study has some limitations. First, CIP was assessed by investigators at each institution. CIP might not be properly categorized. Although it is difficult to differentiate between CIP and RP based on radiographic findings, the frequency of CIP in this study was similar to that in the PACIFIC trial (all-grade CIP, 11.4% [11/96] in our study, 10.7% [51/475] in the PACIFIC trial, and 12.5% [9/72] in the Japanese cohort of the PACIFIC trial)^1^. Therefore, the diagnosis of CIP in this study was reasonable. Second, this was a retrospective study with a limited sample size. Subgroup analyses such as the relationship between dNLR dynamics and survival were performed for small subgroups. Therefore, larger prospective studies are required to confirm our findings.

In conclusion, baseline dNLR helps predict the incidence of irAEs and CIP due to durvalumab in patients with unresectable locally advanced NSCLC who receive durvalumab consolidation therapy following CRT. Moreover, dNLR 28 days after the initiation of durvalumab consolidation therapy and its decreasing changes during treatment are associated with favorable clinical outcomes.

### Supplementary Information

Supplementary Information 1.

Supplementary Figure 1.

Supplementary Figure 2.

Supplementary Figure 3.

Supplementary Figure 4.

Supplementary Figure 5.

Supplementary Figure 6.

## Data Availability

The datasets generated and/or analyzed during the current study are available from the corresponding author on reasonable request.
