# Supplementary material for: Derived neutrophil-to-lymphocyte ratio has the potential to predict safety and outcomes of durvalumab after chemoradiation in non-small cell lung cancer
Source: Sci Rep. 2024 Aug 23;14:19596. doi: 10.1038/s41598-024-70214-y (PMC11343745; doi:10.1038/s41598-024-70214-y)
Supplement: Supplementary file 1 — Supplementary Information 1. [file 41598_2024_70214_MOESM1_ESM.docx]

**Supplementary Material**

**Supplementary Fig. 1** CONSORT diagram

NSCLC, non-small cell lung cancer; CRT, chemoradiotherapy; non-D group, non-durvalumab group; D group, durvalumab group

**Supplementary Fig. 2** Cumulative incidence of grade ≥2 pneumonitis

non-D group, non-durvalumab group; D group, durvalumab group

**Supplementary Fig. 3** Association between dNLR and area of pneumonitis

dNLR, derived neutrophil-to-lymphocyte ratio

**Supplementary Fig. 4** Association between dNLR and irAEs

1. dNLR, and (B) dNLR28 in patients who did and did not experience irAEs.

dNLR, derived neutrophil-to-lymphocyte ratio; irAEs, immune-related adverse events

**Supplementary Fig. 5** Survival effect for dNLR in the non-D group

(A) progression-free survival and (B) overall survival in patients with dNLR ≤ 3 or > 3.

(C) progression-free survival and (D) overall survival in patients with dNLR28 ≤ 3 or > 3.

dNLR, derived neutrophil-to-lymphocyte ratio; non-D group, non-durvalumab group; NA, not applicable

**Supplementary Fig. 6** Alluvial plot of dNLR at baseline and dNLR28

dNLR, derived neutrophil-to-lymphocyte ratio

Supplementary Table 1 Dosimetric data in safety analysis dataset

| Factor |  | non-D group (n = 32) | D group (n = 96) |
| --- | --- | --- | --- |
| Radiation method, n (%) | 3D-CRT | 32 (100) | 75 (78.1) |
|  | IMRT | 0 (0) | 21 (21.9) |
| Irradiated field, n (%) | ENI | 22 (68.8) | 32 (33.3) |
|  | IFRT | 10 (31.2) | 64 (66.7) |
| MLD, median (range), Gy | | 14.49 (4.45-18.51) | 12.88 (2.69-21.12) |
| V5, median (range), % | | 39.52 (14.30-60.87) | 39.37 (8.00-69.28) |
| V20, median (range), % | | 25.44 (6.58-35.24) | 22.97 (4.26-38.47) |
| VS5, median (range), cc | | 1852.5 (1016-4073) | 1905.43 (553-4364) |
| non-D group, non-durvalumab group; D group, durvalumab group; 3D-CRT, three-dimensional conformal radiotherapy; IMRT, intensity-modulated radiotherapy; ENI, elective nodal irradiation; IFRT, involved field radiotherapy; MLD, mean lung dose; V5, lung volume receiving a dose of ≥5 Gy; V20, lung volume receiving a dose of ≥20 Gy; VS5, lung volume spared from 5 Gy. | | | |

Supplementary Table 2 Immune-related adverse events (irAE)

|  | D group (n = 96) | |
| --- | --- | --- |
| Events | Any Grade, n | Grade 3-5, n |
| Pneumonitis | 11 | 3 |
| Hypothyroidism | 10 | 0 |
| Rash | 8 | 0 |
| Infusion reaction | 6 | 0 |
| Liver disorder | 2 | 0 |
| Adrenocortical insufficiency | 2 | 0 |
| Hyperthyroidism | 2 | 0 |
| Heart failure | 1 | 1 |
| Myositis | 1 | 1 |
| Thrombocytopenia | 1 | 1 |
| Acute kidney injury | 1 | 1 |
| Arthritis | 1 | 0 |
| Psoriasis vulgaris | 1 | 0 |
| Diarrhea | 1 | 0 |
| Mucositis oral | 1 | 0 |
| D group, durvalumab group | | |

Supplementary Table 3 Detail of pneumonitis

|  | non-D group (n = 32) | D group (n = 96) | |
| --- | --- | --- | --- |
| Pneumonitis | All, n | All, n | irAE (CIP), n |
| Grade 1 | 27 | 55 | 1 |
| Grade 2 | 2 | 29 | 7 |
| Grade 3 | 0 | 5 | 3 |
| Grade 4 | 0 | 0 | 0 |
| Grade 5 | 1 | 0 | 0 |
| Any Grade | 30 | 89 | 11 |
| non-D group, non-durvalumab group; D group, durvalumab group; irAE, immune-related adverse event; CIP, checkpoint inhibitor-related pneumonitis | | | |

Supplementary Table 4 Univariable analysis for grade ≥2 pneumonitis and CIP

|  | Grade ≥2 pneumonitis | | | | CIP | |
| --- | --- | --- | --- | --- | --- | --- |
|  | non-D group (n = 32) | | D group (n = 96) | | D group (n = 96) | |
|  | OR (95% CI) | *p* value | OR (95% CI) | *p* value | OR (95% CI) | *p* value |
| Age | 1.11 (0.91-1.35) | 0.279 | 1.02 (0.97-1.07) | 0.419 | 1.03 (0.95-1.11) | 0.405 |
| Sex |  | 0.935 |  | 0.026 |  | 0.114 |
| Male | Ref. |  | Ref. |  | Ref. |  |
| Female | 1.11 (0.08-13.9) |  | 0.31 (0.11-0.87) |  | 0.18 (0.02-1.50) |  |
| Smoking history |  | - |  | 0.454 |  | 0.959 |
| Never | Ref. |  | Ref. |  | Ref. |  |
| Former or current | - | - | 1.54 (0.49-4.76) |  | 1.04 (0.20-5.30) |  |
| Emphysema |  | 0.935 |  | 0.338 |  | 0.576 |
| No | Ref. |  | Ref. |  | Ref. |  |
| Yes | 0.94 (0.26-3.35) |  | 0.81 (0.53-1.24) |  | 0.83 (0.45-1.56) |  |
| Histology |  | 0.552 |  | 0.544 |  | 0.540 |
| Non-Sq | Ref. |  | Ref |  | Ref. |  |
| Sq | 2.14 (0.17-26.3) |  | 1.30 (0.560-3.00) |  | 1.48 (0.42-5.24) |  |
| PD-L1 (cutoff: 1%) |  | - |  | 0.824 |  | 0.655 |
| <1 | Ref. |  | Ref. |  | Ref. |  |
| ≥1 | - | - | 0.89 (0.34-2.33) |  | 1.39 (0.03-5.86) |  |
| PD-L1 (cutoff: 50%) |  | - |  | 0.553 |  | 0.446 |
| <50 | Ref. |  | Ref. |  | Ref. |  |
| ≥50 | - | - | 0.70 (0.21-2.27) |  | 1.78 (0.40-7.84) |  |
| Radiation method |  | <0.001 |  | 0.084 |  | 0.298 |
| 3D-CRT | Ref. |  | Ref. |  | Ref. |  |
| IMRT | 0.10 (0.03-0.34) |  | 0.35 (0.10-1.15) |  | 0.32 (0.03-2.70) |  |
| Irradiation field |  | 0.935 |  | 0.763 |  | 0.103 |
| ENI | Ref. |  | Ref. |  | Ref. |  |
| IFRT | 1.11 (0.08-13.9) |  | 0.87 (0.36-2.11) |  | 5.74 (0.70-47.0) |  |
| V5 | 1.14 (0.96-1.34) | 0.120 | 1.04 (1.00-1.08) | 0.030 | 0.98 (0.93-1.03) | 0.539 |
| V20 | 1.32 (0.93-1.86) | 0.113 | 1.07 (1.01-1.14) | 0.025 | 0.94 (0.87-1.03) | 0.207 |
| VS5 | 0.99 (0.99-1.00) | 0.216 | 1.00 (0.99-1.00) | 0.334 | 1.00 (0.99-1.00) | 0.760 |
| MLD | 1.95 (0.79-4.79) | 0.146 | 1.13 (1.01-1.26) | 0.038 | 0.92 (0.79-1.07) | 0.288 |
| dNLR | 0.29 (0.02-3.35) | 0.327 | 0.79 (0.53-1.19) | 0.271 | 0.30 (0.10-0.84) | 0.022 |
| CIP, checkpoint inhibitor-related pneumonitis, non-D group, non-durvalumab group; D group, durvalumab group; CI, confidence interval; OR, odds ratio; Ref. reference; Non-Sq, non-squamous cell carcinoma; Sq, squamous cell carcinoma; PD-L1, programmed death-ligand 1; 3D-CRT, three-dimensional conformal radiotherapy; IMRT, intensity-modulated radiotherapy, ENI, elective nodal irradiation; IFRT, involved field radiotherapy; V5, lung volume receiving a dose of ≥5 Gy; V20, lung volume receiving a dose of ≥20 Gy; VS5, lung volume spared from 5 Gy; MLD, mean lung dose; NLR, neutrophil-to-lymphocyte ratio; dNLR, derived neutrophil-to-lymphocyte ratio. | | | | | | |

Supplementary Table 5 Univariable and multivariable analysis for irAE

| irAE | D group (n=96) | | | |
| --- | --- | --- | --- | --- |
|  | Univariate analysis | | Multivariate analysis | |
|  | OR (95% CI) | *p* value | OR (95% CI) | *p* value |
| Age | 0.98 (0.93-1.03) | 0.439 | - | - |
| Sex |  | 0.021 |  | 0.024 |
| Male | Ref. |  | Ref. |  |
| Female | 0.32 (0.12-0.84) |  | 0.31 (0.11-0.86) |  |
| Smoking history |  | 0.104 |  | - |
| Never | Ref. |  | - |  |
| Former or current | 2.70 (0.81-8.96) |  | - |  |
| Emphysema |  | 0.349 |  | - |
| No | Ref. |  | - |  |
| Yes | 1.22 (0.80-1.85) |  | - |  |
| Histology |  | 0.154 |  | - |
| Non-Sq | Ref. |  | - |  |
| Sq | 0.54 (0.23-1.26) |  | - |  |
| PD-L1 (cutoff: 1%) |  | 0.824 |  | - |
| <1 | Ref. |  | - |  |
| ≥1 | 0.89 (0.34-2.33) |  | - |  |
| PD-L1 (cutoff: 50%) |  | 0.090 |  | - |
| <50 | Ref. |  | - |  |
| ≥50 | 2.64 (0.85-8.10) |  | - |  |
| dNLR | 0.58 (0.36-0.92) | 0.020 | 0.59 (0.11-0.86) | 0.023 |
| D group, durvalumab group; irAE, immune-related adverse event; CI, confidence interval; OR, odds ratio; Ref. reference; Non-Sq, non-squamous cell carcinoma; Sq, squamous cell carcinoma; PD-L1, programmed death-ligand 1; dNLR, derived neutrophil-to-lymphocyte ratio. | | | | |

Supplementary Table 6 Univariate and multivariate analysis for PFS

| PFS | D group (n = 85) | | | |
| --- | --- | --- | --- | --- |
|  | Univariate analysis | | Multivariate analysis | |
|  | HR (95% CI) | *p* value | HR (95% CI) | *p* value |
| Age | 1.02 (0.99-1.06) | 0.147 | - | - |
| Sex |  | 0.769 |  | - |
| Male | Ref. |  | - |  |
| Female | 1.09 (0.59-2.01) |  | - |  |
| Smoking history |  | 0.333 |  | - |
| Never | Ref. |  | - |  |
| Former or current | 0.70 (0.34-1.43) |  | - |  |
| Emphysema |  | 0.846 |  | - |
| No | Ref. |  | - |  |
| Yes | 0.944 (0.52-1.69) |  | - |  |
| Histology |  | 0.020 |  | 0.067 |
| Non-Sq | Ref. |  | Ref. |  |
| Sq | 2.00 (1.11-3.60) |  | 1.84 (0.95-3.56) |  |
| PD-L1 (cutoff: 1%) |  | 0.715 |  | - |
| <1 | Ref. |  | - |  |
| ≥1 | 0.88 (0.46-1.69) |  | - |  |
| PD-L1 (cutoff: 50%) |  | 0.030 |  | 0.047 |
| <50 | Ref. |  | Ref. |  |
| ≥50 | 0.20 (0.04-0.86) |  | 0.23 (0.05-0.98) |  |
| dNLR28 |  | 0.020 |  | 0.044 |
| >3 | Ref. |  | Ref. |  |
| ≤3 | 0.44 (0.22-0.88) |  | 0.47 (0.22-0.98) |  |
| PFS, progression-free survival; D group, durvalumab group; CI, confidence interval; HR, hazard ratio; Ref. reference; Non-Sq, non-squamous cell carcinoma; Sq, squamous cell carcinoma; PD-L1, programmed death-ligand 1; dNLR, derived neutrophil-to-lymphocyte ratio. | | | | |

Supplementary Table 7 Univariate and multivariate analysis for OS

| OS | D group (n = 85) | | | |
| --- | --- | --- | --- | --- |
|  | Univariate analysis | | Multivariate analysis | |
|  | HR (95% CI) | *p* value | HR (95% CI) | *p* value |
| Age | 1.02 (0.97-1.07) | 0.363 | - | - |
| Sex |  | 0.913 |  | - |
| Male | Ref. |  | - |  |
| Female | 1.04 (0.44-2.45) |  | - |  |
| Smoking history |  | 0.419 |  | - |
| Never | Ref. |  | - |  |
| Former or current | 0.68 (0.27-1.72) |  | - |  |
| Emphysema |  | 0.525 |  | - |
| No | Ref. |  | - |  |
| Yes | 1.30 (0.57-2.98) |  | - |  |
| Histology |  | 0.002 |  | 0.004 |
| Non-Sq | Ref. |  | Ref. |  |
| Sq | 4.09 (1.66-10.09) |  | 3.72 (1.49-9.24) |  |
| PD-L1 (cutoff: 1%) |  | 0.810 |  | - |
| <1 | Ref. |  | - |  |
| ≥1 | 0.89 (0.35-2.22) |  | - |  |
| PD-L1 (cutoff: 50%) |  | - |  | - |
| <50 | Ref. |  | - |  |
| ≥50 | -* |  | - |  |
| dNLR28 |  | 0.037 |  | 0.131 |
| >3 | Ref. |  | Ref. |  |
| ≤3 | 0.39 (0.16-0.94) |  | 0.50 (0.20-1.22) |  |
| OS, overall survival; D group, durvalumab group; CI, confidence interval; HR, hazard ratio; Ref. reference; Non-Sq, non-squamous cell carcinoma; Sq, squamous cell carcinoma; PD-L1, programmed death-ligand 1; dNLR, derived neutrophil-to-lymphocyte ratio.  * HR cannot be calculated because no events were occurred in PD-L1 ≥50 group. | | | | |
