# Supplementary figures and images for: Derived neutrophil-to-lymphocyte ratio has the potential to predict safety and outcomes of durvalumab after chemoradiation in non-small cell lung cancer
Source: Sci Rep. 2024 Aug 23;14:19596. doi: 10.1038/s41598-024-70214-y (PMC11343745; doi:10.1038/s41598-024-70214-y)

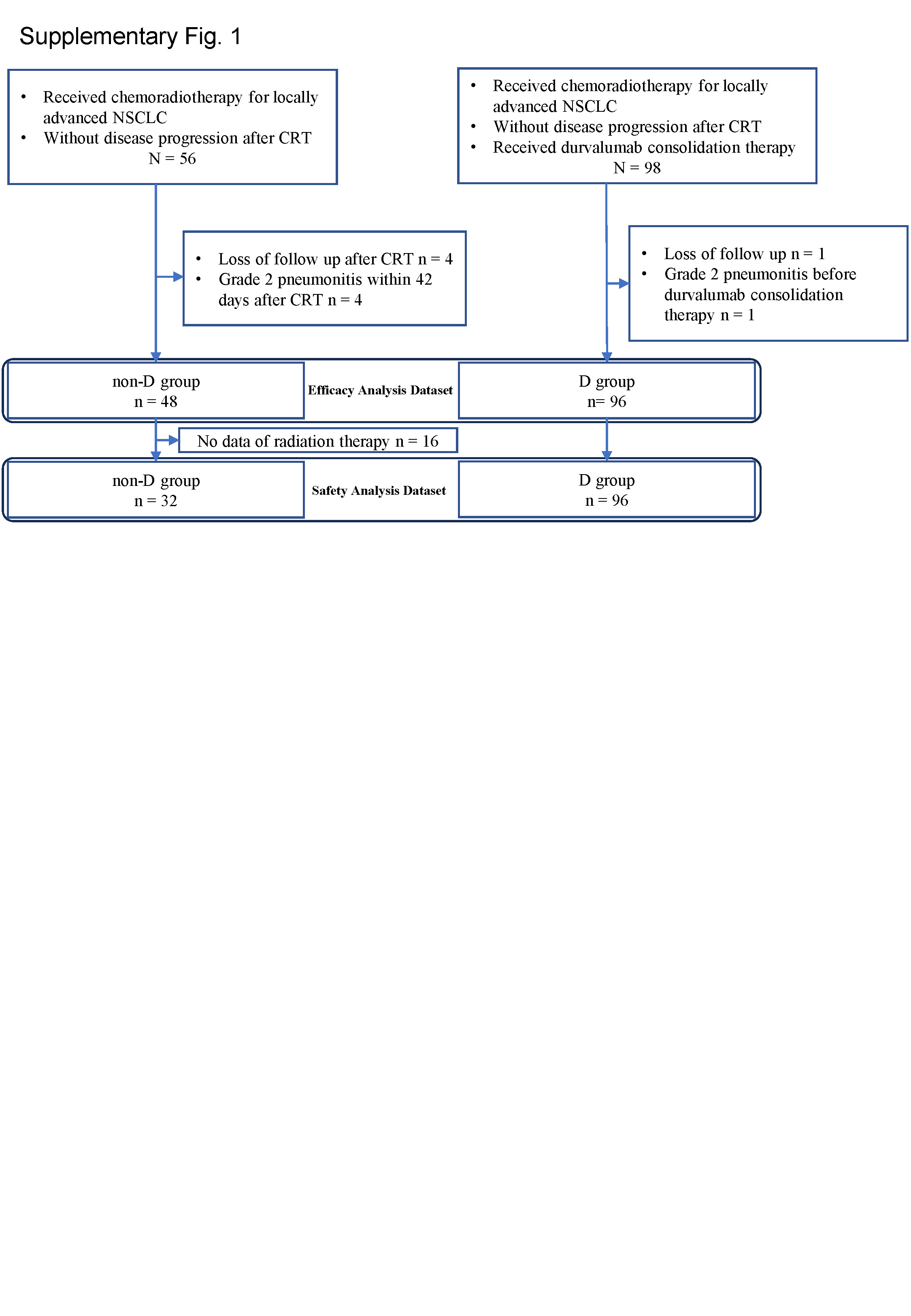

Supplement: Supplementary file 2 — Supplementary Figure 1. [file 41598_2024_70214_MOESM2_ESM.jpg]

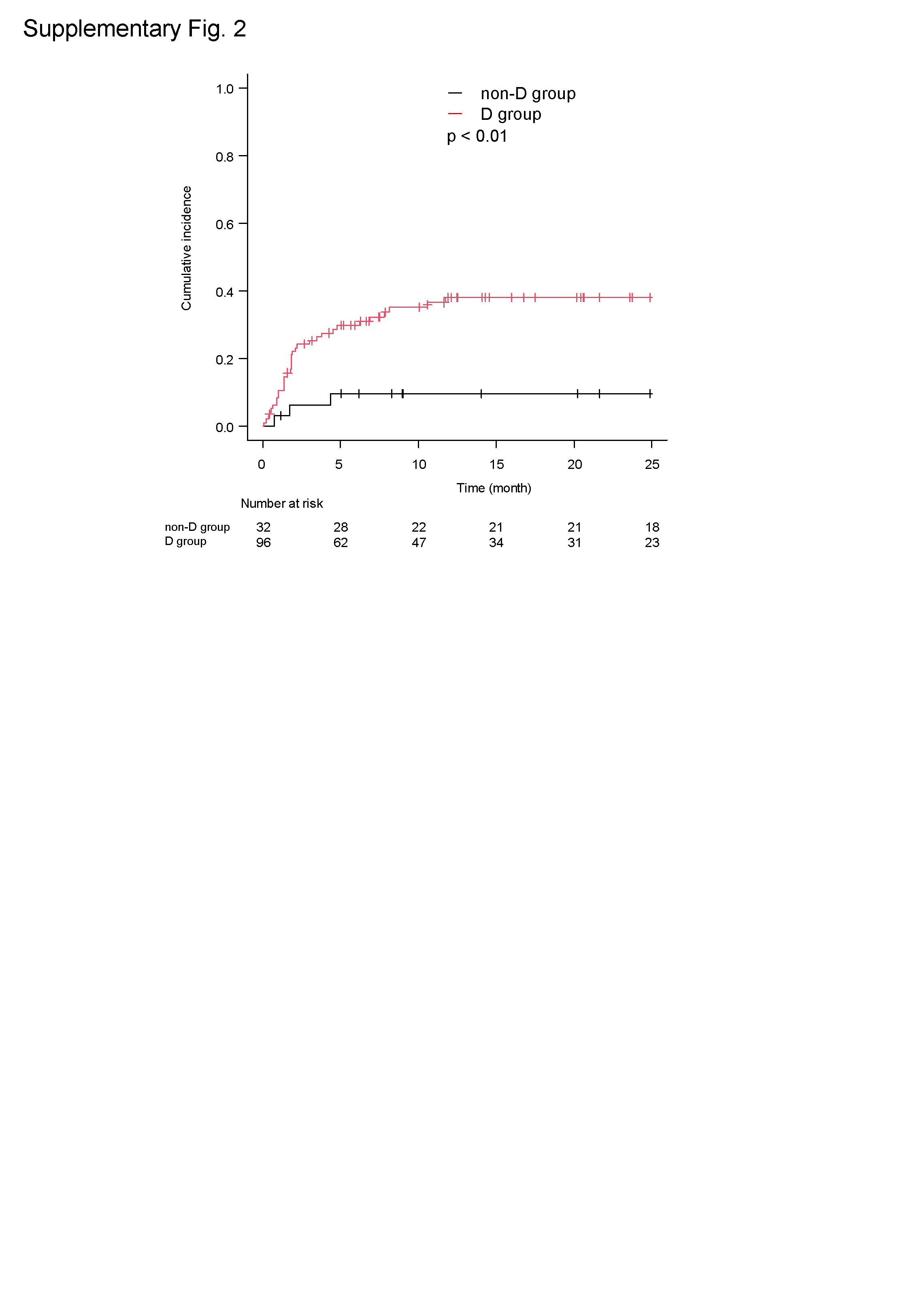

Supplement: Supplementary file 3 — Supplementary Figure 2. [file 41598_2024_70214_MOESM3_ESM.jpg]

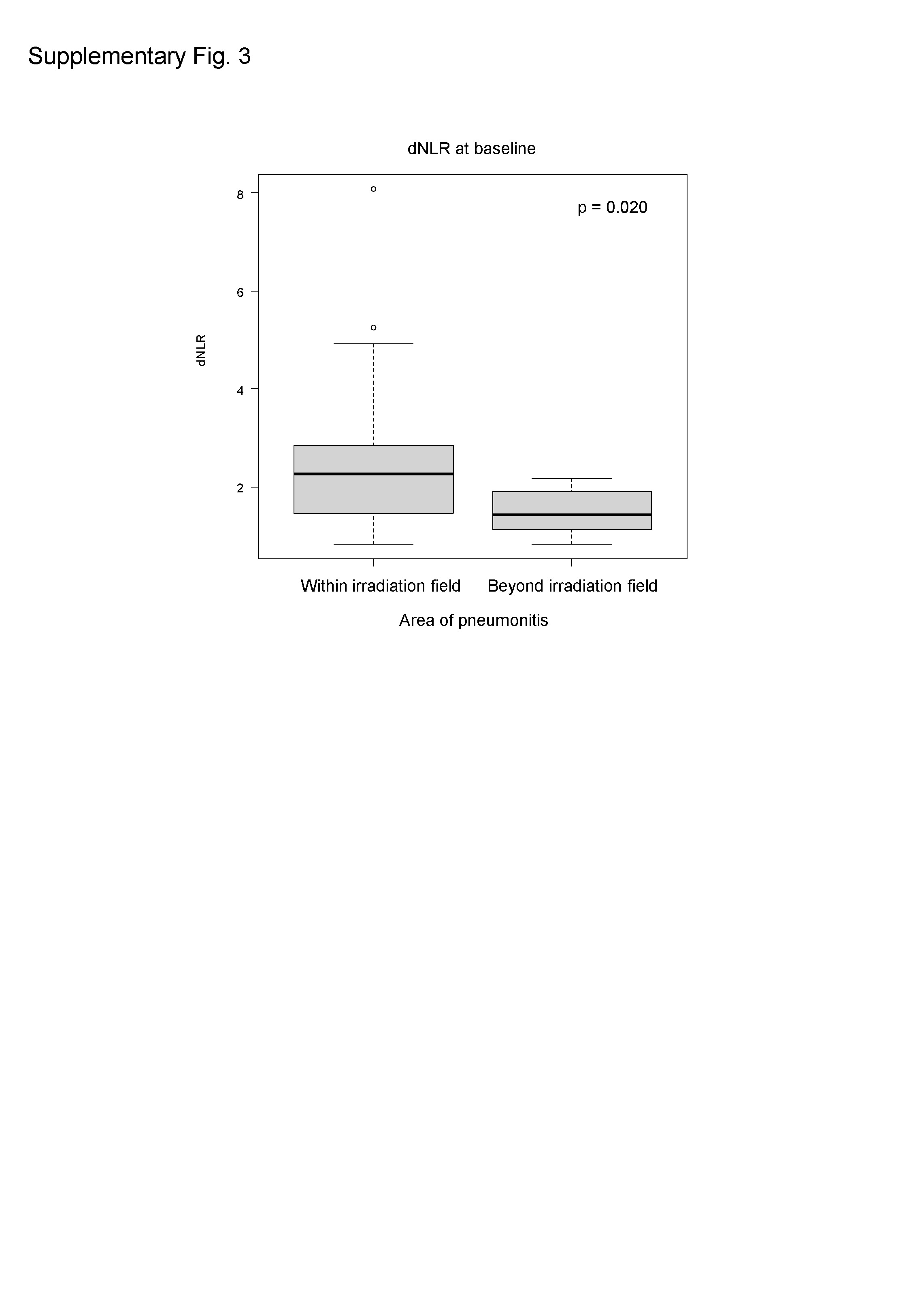

Supplement: Supplementary file 4 — Supplementary Figure 3. [file 41598_2024_70214_MOESM4_ESM.jpg]

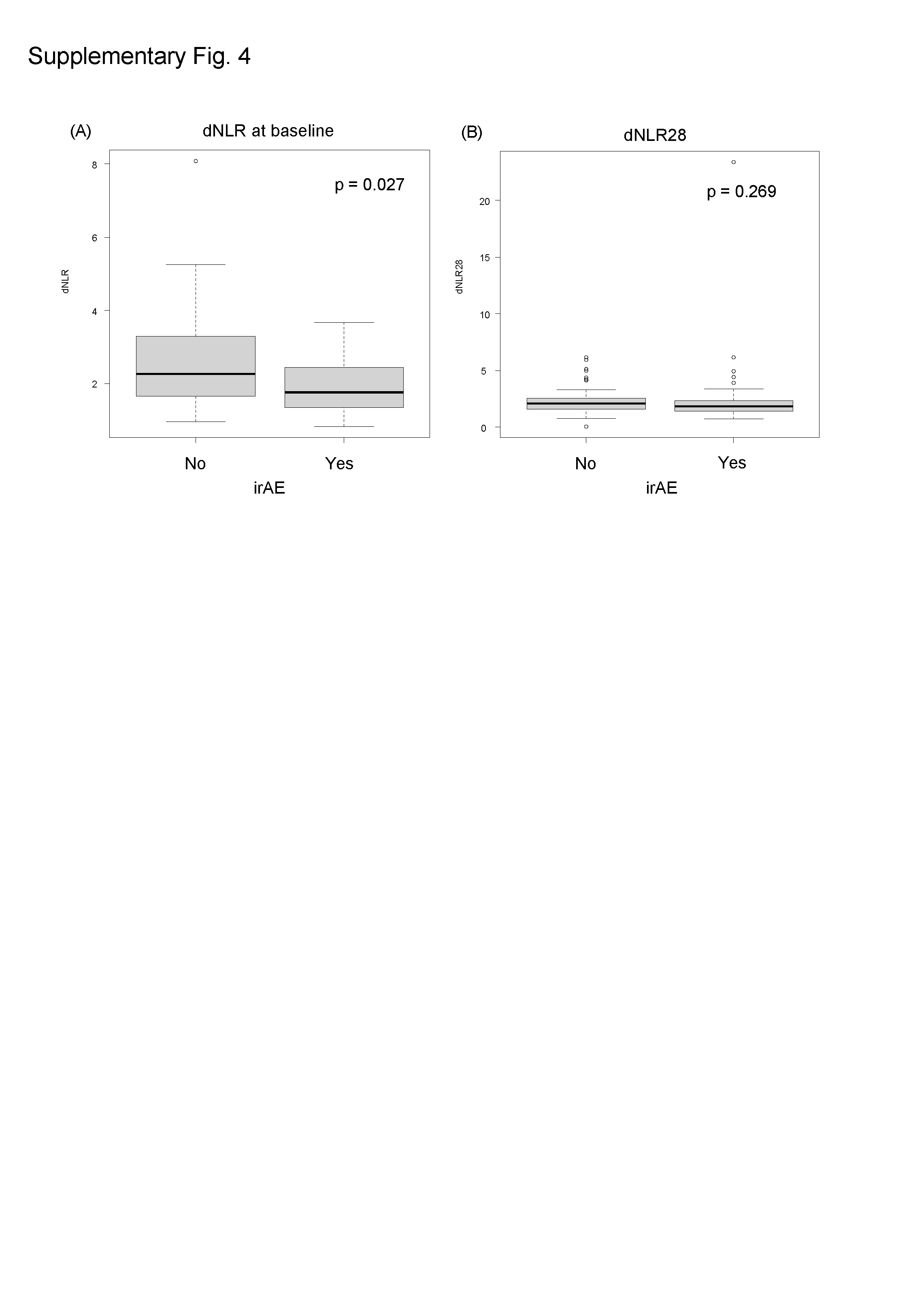

Supplement: Supplementary file 5 — Supplementary Figure 4. [file 41598_2024_70214_MOESM5_ESM.jpg]

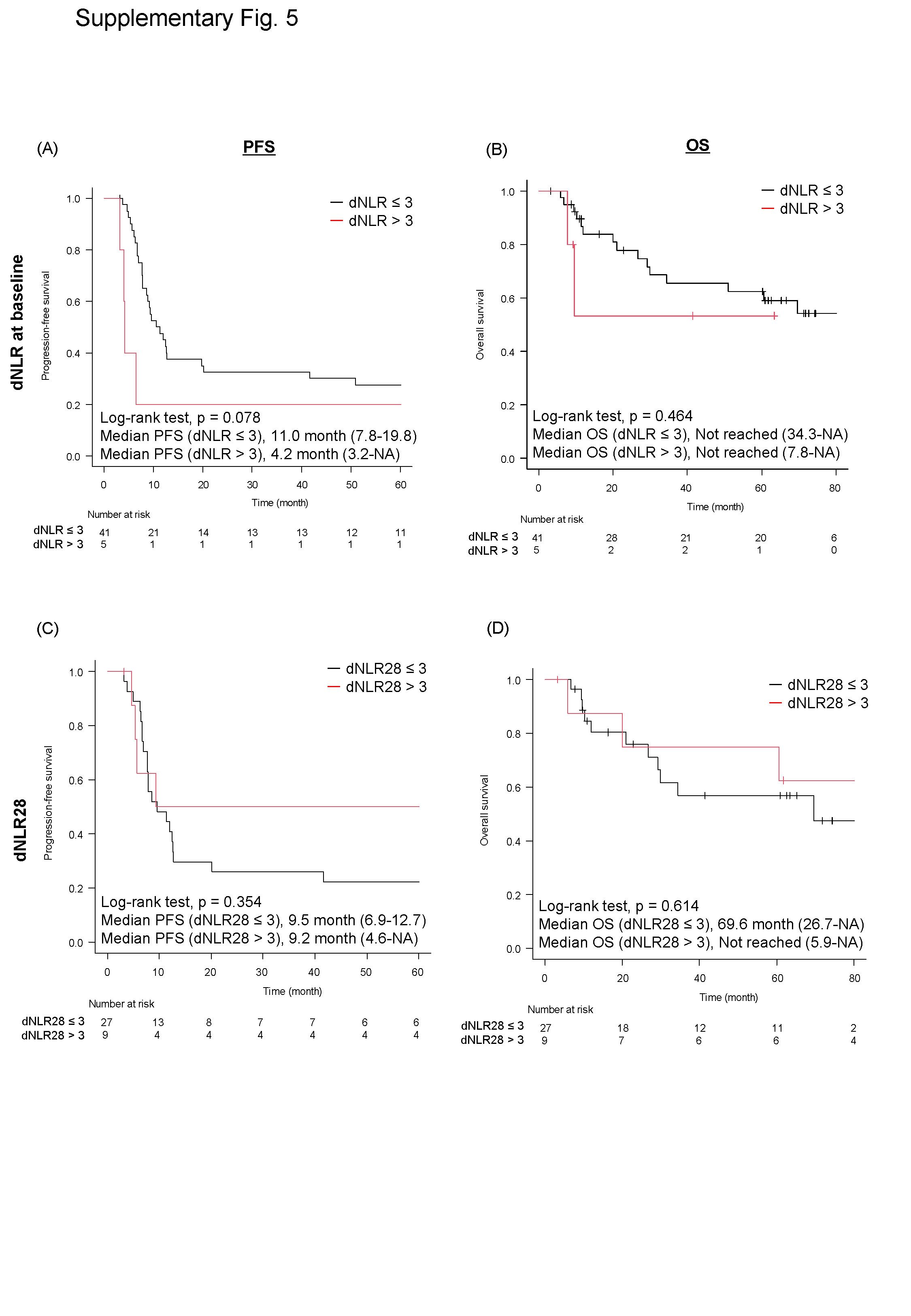

Supplement: Supplementary file 6 — Supplementary Figure 5. [file 41598_2024_70214_MOESM6_ESM.jpg]

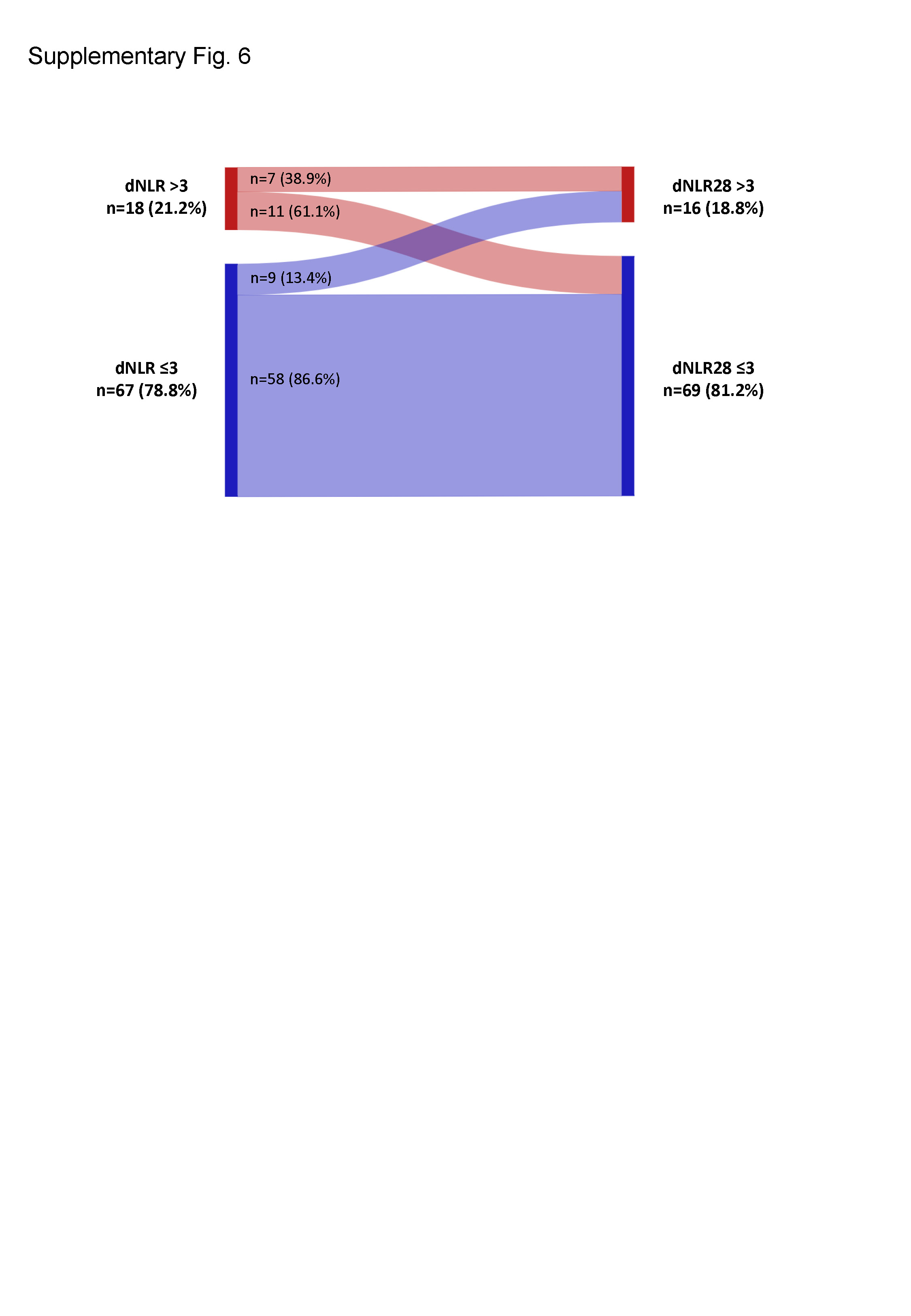

Supplement: Supplementary file 7 — Supplementary Figure 6. [file 41598_2024_70214_MOESM7_ESM.jpg]
